# II. Capsular vaso-mimicry formed by transgenic mammary tumor spheroids implanted ectopically into mouse dorsal skin fold: implications for cellular mechanisms of metastasis

**DOI:** 10.12688/f1000research.2-9.v2

**Published:** 2013-04-23

**Authors:** Halina Witkiewicz, Phil Oh, Jan E Schnitzer

**Affiliations:** 1 Proteogenomics Research Institute for Systems Medicine, San Diego, CA, 92121, USA

## Abstract

Most cancer patients die of metastatic disease, not primary tumors, while biological mechanisms leading to metastases remain unclear and effective therapies are missing. Using a mouse dorsal skin chamber model we had observed that tumor growth and vasculature formation could be influenced by the way *in vitro* cultured (avascular) spheroids of N202 breast tumor cells were implanted; co-implantation of lactating breast tissue created stimulating microenvironment, whereas the absence of the graft resulted in temporary tumor dormancy. This report addressed the issue of cellular mechanisms of the vasculogenic switch that ended the dormancy. *In situ* ultrastructural analysis revealed that the tumors survived in ectopic microenvironment until some of host and tumor stem cells evolved independently into cells initiating the vasculogenic switch. The tumor cells that survived and proliferated under hypoxic conditions for three weeks were supported by erythrogenic autophagy of others. However, the host microenvironment first responded as it would to non-immunogenic foreign bodies, i.e., by encapsulating the tumor spheroids with collagen-producing fibroblasts. That led to a form of vaso-mimicry consisting of tumor cells amid tumor-derived erythrosomes (synonym of erythrocytes), megakaryocytes and platelets, and encapsulating them all, the host fibroblasts. Such capsular vaso-mimicry could potentially facilitate metastasis by fusing with morphologically similar lymphatic vessels or veins. Once incorporated into the host circulatory system, tumor cells could be carried away passively by blood flow, regardless of their genetic heterogeneity. The fake vascular segment would have permeability properties different from genuine vascular endothelium. The capsular vaso-mimicry was different from vasculogenic mimicry earlier observed in metastases-associated malignant tumors where channels formed by tumor cells were said to contain circulating blood. Structures similar to the vasculogenic mimicry were seen here as well but contained non-circulating erythrosomes formed between tumor nodules. The host’s response to the implantation included coordinated formation of new vessels and peripheral nerves.

***"Discovery consists of seeing what everybody has seen and thinking what nobody has thought".***

*– Albert Szent-Gyorgyi *

## Introduction

Two main problems persisting in oncology are related; they are (1) the incomplete understanding of the mechanism by which tumors spread from primary locations to multiple organs and (2) the lack of selective anti-cancer treatments. A developmental regulatory program involved in embryo implantation, referred to as the "epithelial-mesenchymal transition" (EMT) ^1– 4^ was adopted to explain how transformed epithelial cells could acquire ability to metastasize, i.e., to invade surrounding nonmalignant tissues and to disseminate, in a multistep process including entering and leaving the circulatory system ^5, 6^. However, no satisfactory mechanism for the spreading of non-epithelial tumors to secondary locations was proposed. Therefore, an alternative to EMT regulatory programs playing a role in invasiveness of carcinoma cells should also be considered, as pointed out elsewhere ^7^.

Attempts made to elucidate the cellular mechanism of metastasis-initiating events included retrospective extrapolation from the distribution of established metastases, namely the preference of specific tumors to metastasize in certain organs but not in others. One of the earliest studies addressed the issue by injecting fixed and stained tumor cells into the left side of the heart in rabbits and determining their subsequent distribution in tissue sections of several organs. The results supported the hypothesis that the distribution of metastases was determined by the mechanics of circulation and the consequent location of embolic tumor cells but they did not exclude a role of local "soil" factors ^8^. Other studies, by making a connection with genetic diversity of tumors, suggested that metastases might have a clonal origin and the dissociated representatives of particular subpopulations could be directed to their related tissues. However, generating biological diversity continued among different metastatic foci ^9^. Metastases of particularly aggressive cancers of different types (ovarian ^10, 11^, prostate ^12, 13^, glioblastoma ^14^, as well as melanoma ^15^) were associated with patterned vasculogenic mimicry, i.e., a network of periodic acid Schiff stained (glycoproteins containing ^16^) "loops" that represented blood-containing micro-vascular "channels", generated by the aggressive tumor cells without participation of endothelial cells (ECs) and independently of angiogenesis ^17, 18^. How these structures facilitated metastasis was not clear ^19, 20^. Elevated incidence of metastasis was also correlated with autophagy of internal organelles in tumor cells, although by what mechanism was not clear ^21^. Reports based on different experiments suggested that depending on the context, autophagy could either stimulate or prevent cancer ^22^. Thus, the question regarding the way in which autophagy influenced metastasis remained unanswered ^23^. Two other intriguing issues were inefficiency of tumor formation in experimental settings and targeting of a selected sub-population of tumor cells by an anticancer drug. (1) Theoretically a single cell could be capable of establishing the tumor but large numbers and a latent period were actually required ^24, 25^. (2) A selective uptake of the endoradiotherapeutic 6-[211 At]-astato-2-methyl-1,4-naphtoquinol bis(diphosphate) drug only by those tumor cell nuclei that contained alkaline phosphatase isoenzyme was demonstrated ^26^. Those observations together with the heterogeneity of tumors, known for a long time but not fully understood ^25, 27^, suggested the existence of cancer stem cells (CSCs) in spite of the undifferentiated phenotype of the malignant cells. The definitive proof of the CSCs was lacking ^28^.

The problem with nonspecific side-effects causing activities of anti-cancer drugs was to be circumvented by targeted drug delivery. That hope was based on the observation that endothelial surfaces had variable, organ-specific properties ^29– 34^. However, crossing the endothelial barrier by molecules that successfully reached the intended vascular destinations turned out to be another problem. The targeted destruction of established tumor vessels themselves resulted only in reduction of the tumor growth ^35^ (as expected by J. Folkman ^36^) because tumors could regenerate their vasculature. The approach did appear effective in some non-neoplastic diseases ^37, 38^. The issue of the tumor vessels permeability is rather perplexing. On one hand the vessels prevent anticancer drugs from penetrating the tumors, and on the other they are known to be abnormally leaky ^39, 40^.

We had observed earlier that in our model formation of the tumor vasculature (vessels and blood) could be accelerated by availability of homologous tissue stem cells (TSCs) from co-implanted graft ^41^. Without them, the process was relatively slow and the growth of implanted avascular tumor spheroids was limited, yet eventually the vasculogenic switch did happen. That raised the following questions: (1) how did the tumor survive the lag period (about three weeks) without vasculature and (2) how was the problem solved eventually? In addition to providing answers to those questions, the results shown below suggested a new cellular mechanism for initiating metastasis. We use the term TSCs according to the 2002 functional definition by M. Loeffler and I. Roeder ^42, 43^. It refers to the stemness as a capability of a system rather than individual cell lineages. We present a new type of vaso-mimicry in the murine model of breast tumor that was morphologically different from the vasculogenic mimicry previously described and postulate its role in facilitating the passive transport of tumor cell clusters to secondary locations and in determining the increased permeability of such fake vessels. Understanding the process is critical from the clinical point of view. If correct, it would bring the focus of the future studies to the energy metabolism-related initial steps (as discussed elsewhere ^41^) and could result in finding new ways for inhibiting some of them before the angiogenic switch has had a chance to evolve; therefore, potentially preventing the metastases.

## Materials and methods

The study was performed according to protocols approved by Sidney Kimmel Cancer Center’s OLAW-approved Institutional Animal Care and Use Committee (Assurance No A4128-01). The protocol numbers were: 03-16A and 05-11 for Grants CA104898 and CA119378, respectively. No human specimens were involved in any of the experiments outlined here.

Two recipient mice of the 5 used in the two accompanying articles were assessed in this experiment. The same numbering system was used in all three articles. The experimental design is summarized in Table 1.

**Table 1.** Experimental design.

Figure numbers	Mouse no	Mouse strain	Tumor cell line	Graft tissue	Incubation time (days)
1 (A & C–I), 2 (B–F), 4 (A–D), 5	4	AN/NU	N202.1A+H2B-GFP	None	22
1 (B), 2 (A), 3	1	AN/NU	N202.1A+H2B-GFP	None	21

### Animals, tumor spheroids, chambers and antibodies

The host mice were 8–9 weeks old athymic nude females purchased from Harlan. They were housed in the SKCC animal care facilitywith controlled 12/12 hr light/dark cycle and temperature maintained at + 22°C. The mice were on Tekand global 14% protein rodent diet (Harlan) with access to water ad libitum. For surgery, they were anesthetized (7.3 mg ketamine hydrochloride and 2.3 mg xylazine/100 g body weight, inoculated i.p.) and placed on a heating pad. Immediately before tissue harvesting the tumor hosting mice as well as the graft donors were euthanized with pentobarbital overdose (100 mg/kg i.p.).

The N202.1A+H2B-GFP cell line (generated by stable transfection of the parental murine breast cancer cell line, N202.1A ^44^ to express GFP under histone H2B promoter ^45^) was obtained from Drs. J. Lustgarten and P. Borgstrom and used to form tumor spheroids by culturing 2×10 ^5^ cells per well for 2–3 days prior to implantation. A week after establishment of mouse dorsal skin chambers, the tumor spheroids were implanted directly on skin (ectopically) ^46^. The tumors were incubated in the chambers for three weeks ( Table 1). Their final size was about 1–3 mm in "diameter'. The GFP-specific rabbit polyclonal IgG (ab290) was from Abcam; and non-reactive IgG (sc-34284) were from Santa Cruz.

### Tissue processing

The tumors with some surrounding tissues were dissected out and cut in halves perpendicularly to the host skin surface while immersed in cold fixative (4% paraformaldehyde in 0.1 M Na cacodylate pH 7.4). The skin region served later as a reference to distinguish between edges of the tumor facing the skin and those facing the glass window of the chamber. The halves were then separated and processed independently for TEM and immunocytochemistry.

### TEM

The tissues were transferred into a stronger fixative (4% paraformaldehyde/2.0% glutaraldehyde in 0.1 M Na cacodylate pH 7.4) to better preserve the ultrastructures before further cutting. They were cut into 1 mm thick slices in planes perpendicular to the plain of the first cut and to the skin surface, finally, into ~ 1 mm ^3^ blocks, transferred into fresh portion of the fixative in which they were cut and incubated for 2 hrs at 4°C. The fixed tissue blocks were washed with 0.1 M Na cacodylate–HCl buffer pH 7.4 (3 × 15 min.) and post fixed in 1% OsO _4_ in 0.1 M Na cacodylate buffer, pH 7.0 for 60 min on ice, washed with water and stained with 1% uranyl acetate at RT for one hour. The blocks were embedded in EMbed-12 (EM Sciences, Cat No. 14120). The resin embedded tissues were cut into 60 nm sections on Leica Ultracut UCT ultramicrotome and viewed without further contrasting.

### Immunocytochemistry

During cutting into ~ 1 mm ^3^ blocks as described above, the tissues were kept in the mild fixative to protect the antigenic epitopes (4% paraformaldehyde in 0.1 M Na cacodylate pH 7.4). The tissue blocks were vitrified by infiltrating the pieces with 50% PVP (polyvinylpyrrolidone) containing 2.3 M sucrose in 0.1 M Na cacodylate buffer, pH 7.4, for 2 hrs or over night, mounted on metal pins and frozen in liquid nitrogen, as described by Tokuyasu ^47^. Frozen tissues were cut into 70 nm sections, on Leica Ultracut UCT ultramicrotome with the cryo-attachment. The sections were picked from the knife with 2.3 M sucrose and floated on 1% ovalbumin (Sigma, Cat No. A5378) in 0.1 M Na cacodylate buffer for at least one hour before incubation with specific or non-reactive antibody (50 µg/ml), at RT for one hour. Sections were then rinsed eight times with 0.1% ovalbumin in the same buffer and incubated for one hour with 10 nm Au coupled to protein A (from Dr G. Posthuma; Cell Microscopy Center, university Medical Center Utrecht, The Netherlands). The eight rinsing steps were repeated before fixation of the immune complexes with 1% glutaraldehyde. After rinsing three times with water the immunostained cryosections were contrasted with mixture of uranyl acetate and methyl cellulose (25 centipoises, Sigma M-6385) in water, at final concentration of 1.3% each, for 10 min at RT. Excess of the liquid was removed and the sections were dried at RT.

### Viewing

All sections were viewed and the images captured at 100 kV using a Morgagni 268 electron microscope equipped with a MegaView III digital camera. Analyzed tissue sections were first examined at low magnification and coordinates for each hexagonal sector of a grid covered with tissue were recorded automatically. Subsequently all sectors were explored at least once at variable high magnifications. Interesting images were captured at the magnification best suited to document a particular phenomenon or identify a structure, including colloidal Au grains. The images were transmitted from the microscope camera to iTEM imaging platform from Olympus Soft Imaging Solutions and archived in a designated Data Base. In some cases the final images were assembled by multiple image alignment (MIA) to increase the surface area without losing the resolution. We used graphics editing program, Adobe PhotoShop, to add cell type specific color-coding shown in the twin set of images included in the Supplement.

## Results

**Figure 1.**
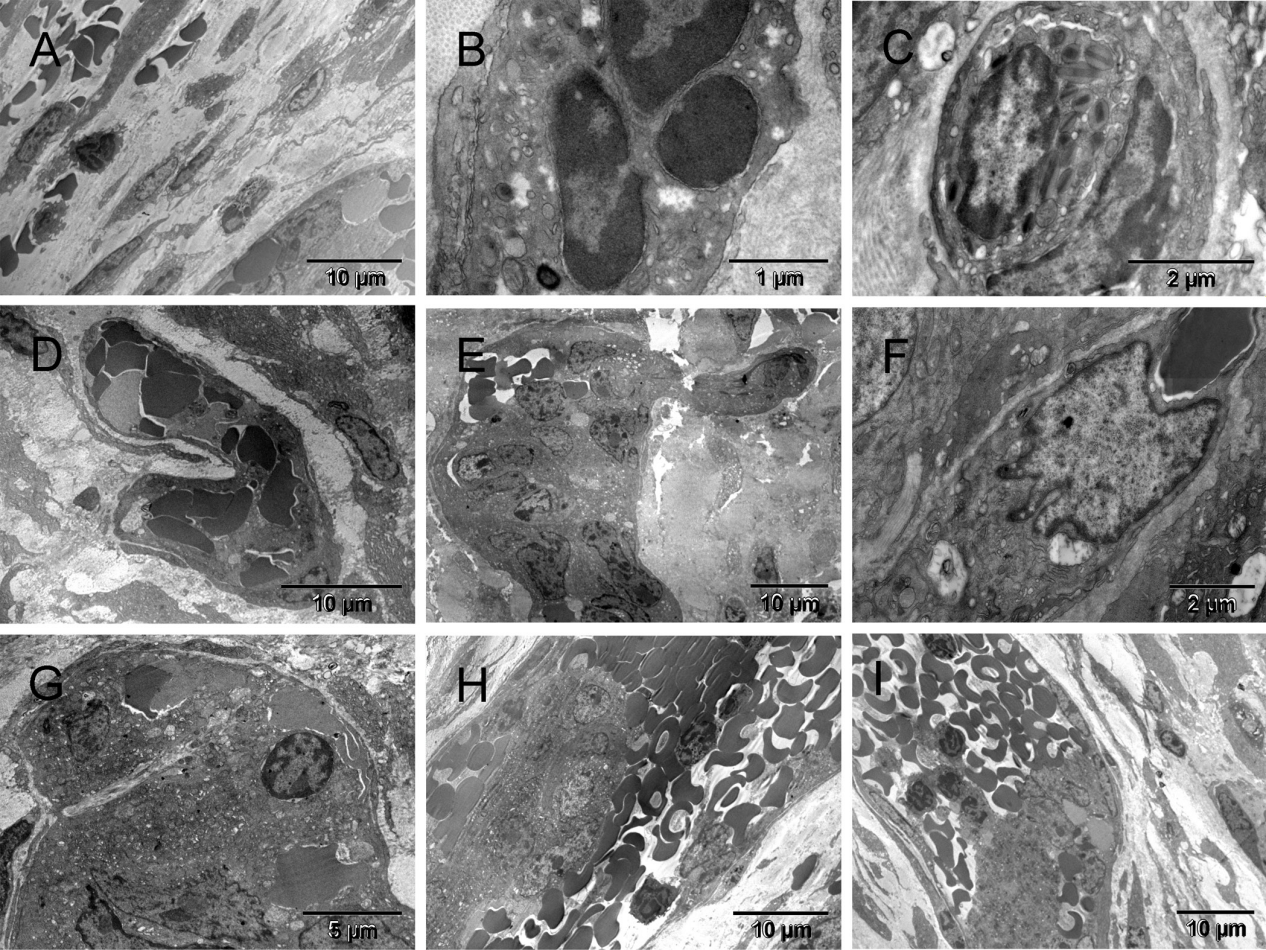
Delay in tumor vasculature development and capsular vaso-mimicry in the ectopic environment. Three weeks after implantation, the tumor environment consisted of single migrating cells that were secreting ECM components, mainly collagen, and others converting into erythrosomes [ **A**]. The erythroblast [ **B**] and the eosinophil [ **C**] were each in contact with a nucleated supporting cell on one side. A small primitive vessel located next to the tumor [ **D**] resembled those early ones seen in pseudo-orthotopic environment five days after implantation (Figure 2 in ^41^). Tumor cells were encapsulated by fibroblasts and the cell population inside the capsules appeared heterogeneous. There were erythrosomes [ **E**] and nuclei of some cells had sinuses [ **F** & **G**] similar to those in ECs of a forming artery (Figure 8 in ^41^). Featured in [ **H**] is a part of larger elongated capsule containing a cluster of tumor cells in the center and multiple erythrosomes around it, all covered with typical flat, collagen producing fibroblasts, whereas a similar structure in [ **I**] contained mainly erythrosomes and platelets. Both were remarkably similar to a vein.

**Figure S1.**
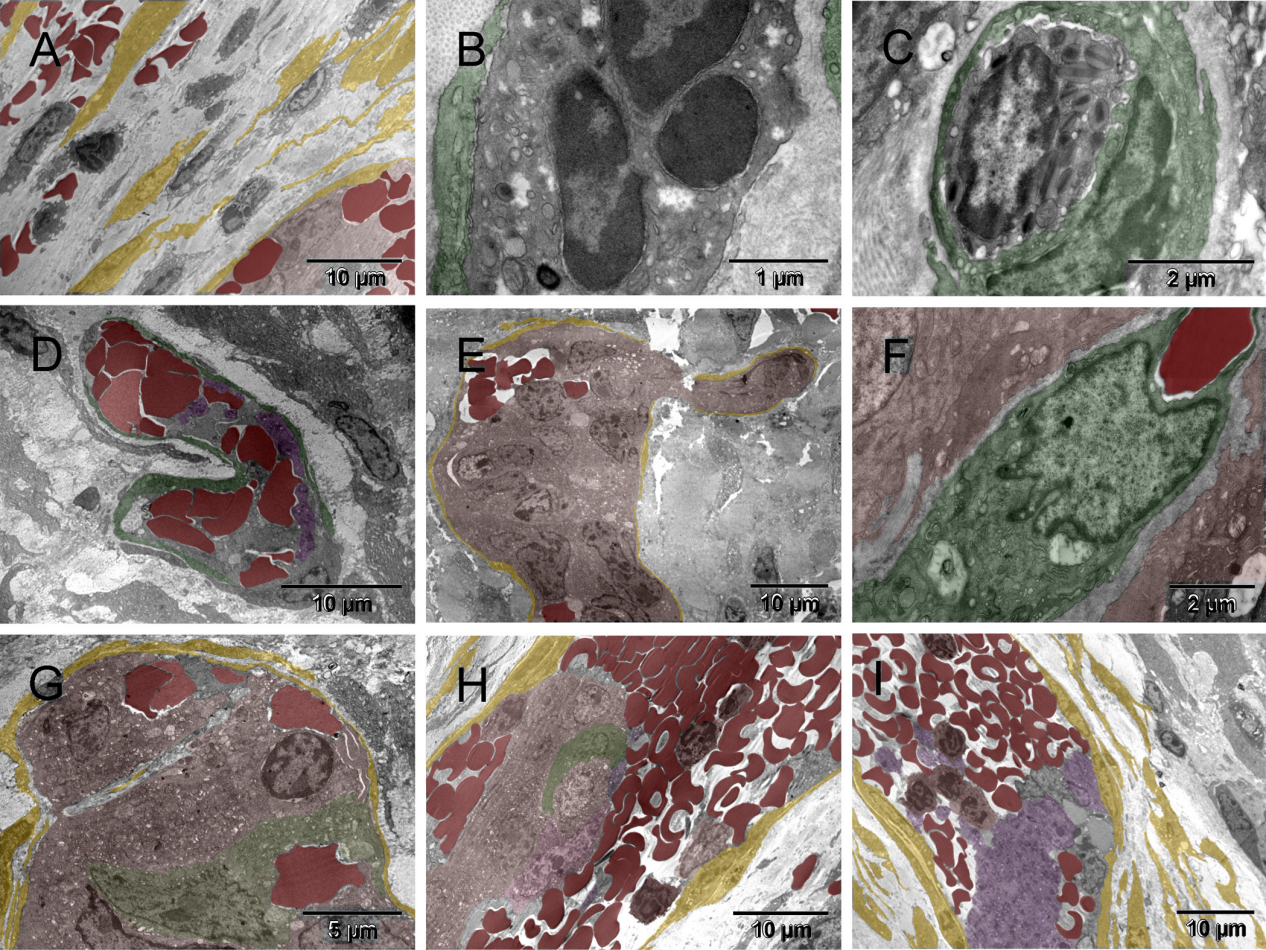
Delay in tumor vasculature development and capsular vaso-mimicry in the ectopic environment. Three weeks after implantation, the tumor environment consisted of single migrating cells that were secreting ECM components, mainly collagen (yellow), and others converting into erythrosomes (red) [ **A**]. The erythroblast [ **B**] and the eosinophil [ **C**] were each in contact with a nucleated supporting cell on one side (green). A small primitive vessel, constituted mainly by erythrosomes (red) held together with megakaryocytes and platelets (purple), was located close to tumor (brown) [ **D**]. It resembled those early ones seen in pseudo-orthotopic environment five days after implantation (Figure 2 in ^41^). Tumor cells were encapsulated by fibroblasts (yellow) and the cell population inside the capsules appeared heterogeneous [ **E**]. There were erythrosomes (red) and nuclei of some cells had sinuses (green) [ **F** & **G**] similar to those in ECs of a forming artery (Figure 8 in ^41^). Featured in [ **H**] is a part of larger elongated capsule containing cluster of tumor cells (brown) in the center and multiple erythrosomes around it, all covered with typical flat, collagen producing fibroblasts, whereas a similar structure in [ **I**] contained mainly erythrosomes and platelets (purple). Both were remarkably similar to a vein.

Three weeks after the ectopic implantation of tumor spheroids, the vasculature formation, i.e., formation of tumor-supporting blood and vessels, was evidently retarded in comparison to pseudo-orthotopically implanted tumors described elsewhere ^41^. However, the host response to the surgical injury was well advanced ( Figure 1 & Figure S1). A multi-cellular layer of connective tissue was growing between tumor and glass wall of the chamber, therefore, it was also generating its own vasculature ([A] in Figure 1 & Figure S1). Here, the term "vasculature" includes vessels and blood, and the term "erythrosome" is used as synonym for the "erythrocyte", because the latter is not a cell ^41^. Acellular areas of collagen matrix contained erythrosomes that were vessel-free, although not extravasated. Those areas were not necrotic. Occasionally some blood cells were in close contact with supporting nucleated cells ([B & C] in Figure 1 & Figure S1). Outside the tumor capsule, a primitive forming vessel morphologically resembled some of those seen around pseudo-orthotopically implanted tumors after only five days ([D] in Figure 1 & Figure S1). Fibroblasts also encapsulated small tumor nodules. The population of tumor cells inside the encapsulated nodules was heterogeneous ([E–G] in Figure 1 & Figure S1). Evidently the tumor cells were also capable of converting into erythrosomes and by doing so in a non-synchronized fashion, they could enable survival of other tumor cells. However, the tumor’s ability to generate the genuine vessels was limited at that stage; therefore, the tumor could not grow. Yet some tumor cells (CSCs?) began regenerating their vasculogenic potential that had been dormant during the years of *in vitro* culturing ([F & G] in Figure 1 & Figure S1). Thus the vasculogenic switch did occur in the absence of the homologous tissue stem cells (TSCs) from the graft but only after a considerable delay (about two weeks). Until that time, some tumor cells survived at the expense of the others.

**Figure 2.**
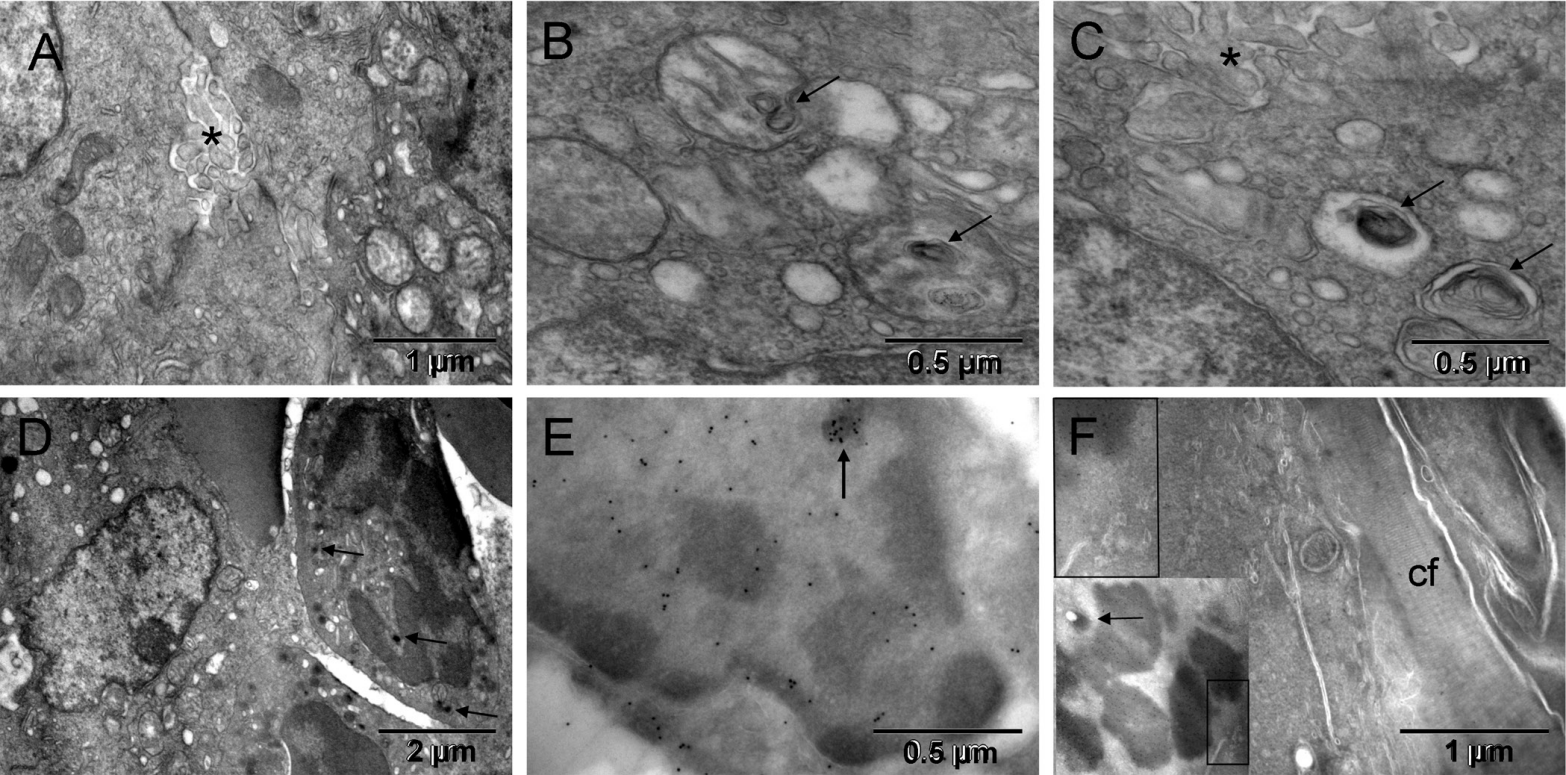
Hypoxia in tumor cells and involvement of host cells in forming the capsular vaso-mimicry. Hypoxia in tumor cells was manifested by structural changes of mitochondria resulting in their condensation into smaller and more electron dense (darker) forms or in degradation of cristae (left and right, respectively) [ **A**], occasionally leading to autophagic vacuoles with whorls of membranes (arrows in [ **B** & **C**]) or to peroxisomes (arrows in [ **D**]). Partial separation of the tumor cells from one another (anoikis ^58^) with emergence of meandering nanotentacles (stars in [ **A** & **C**]) created intercellular passages and greatly increased the cell surface. Immunocytochemical detection of GFP-labeled histone H2B in tumor cells is shown in [ **E** & **F**]. The tumor cell in [ **E**] had a nucleus with electron dense regions that did not contain H2B, except for a small upper region, demonstrating incomplete chromatin degradation (arrow). Upper left corner of [ **F**] corresponded to the boxed area of the insert and showed H2B specific label in mitotic chromosome of the tumor cell. A layer of collagen fibers (cf) separated that cell from the elongated fibroblast with unlabeled nucleus (upper right corner) of [ **F**]. Arrow in the insert points to a condensed mitochondrion.

**Figure S2.**
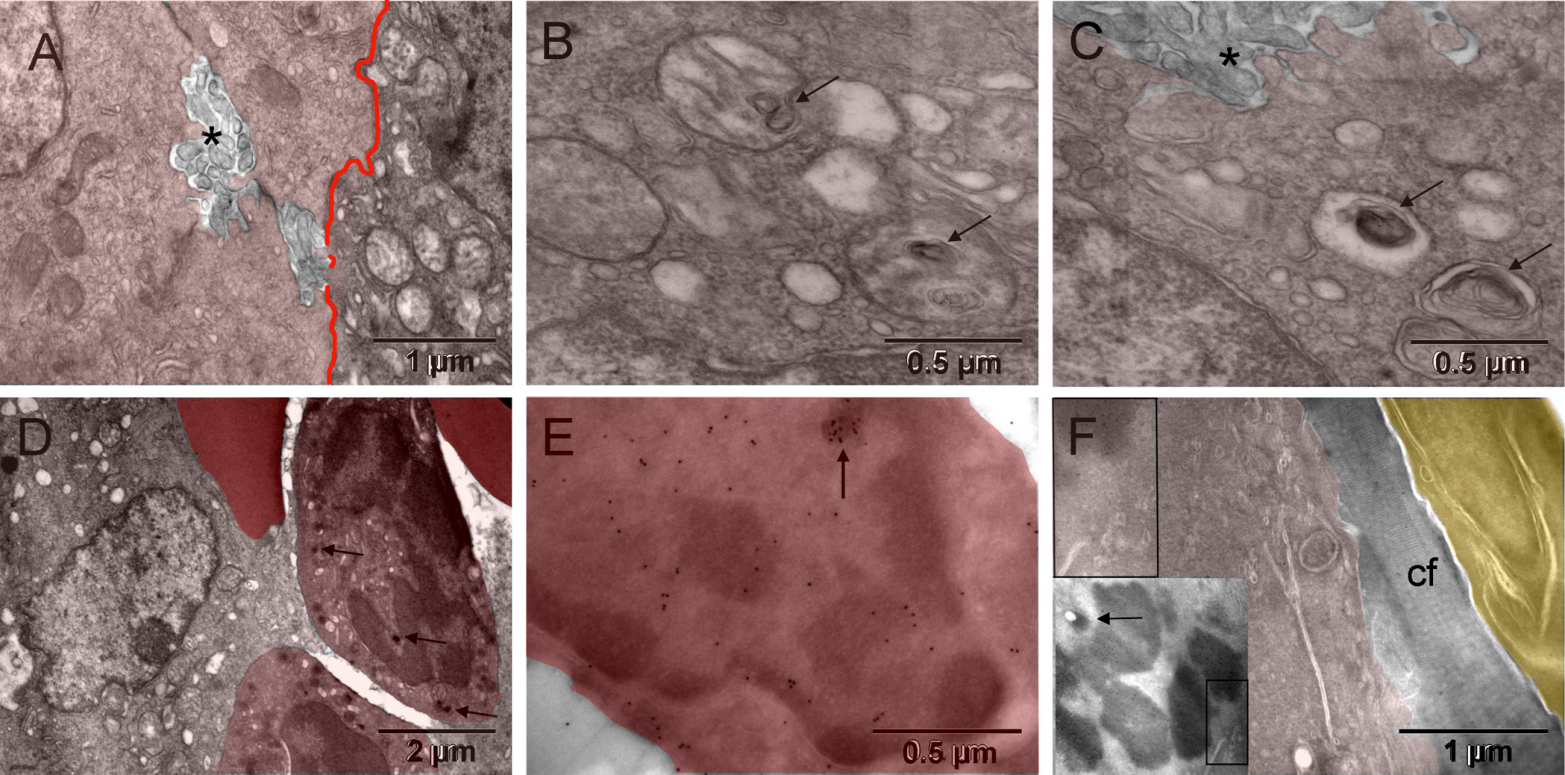
Hypoxia in tumor cells and involvement of host cells in forming the capsular vaso-mimicry. Hypoxia in tumor cells was manifested by structural changes of mitochondria resulting in their condensation into smaller and more electron dense (darker) forms or in degradation of cristae (left and right from the red line, respectively) [ **A**], occasionally leading to autophagic vacuoles with whorls of membranes (arrows in [ **B** & **C**]) or to peroxisomes (arrows in [ **D**]). Partial separation of the tumor cells from one another (anoikis ^58^) with emergence of meandering nanotentacles (stars in [ **A** & **C**], no color added) created intercellular passages and greatly increased the cell surface. Immunocytochemical detection of GFP-labeled histone H2B in tumor cell is shown in [ **E** & **F**]. The tumor cell in [ **E**, light red] had nucleus with electron dense regions that did not contain H2B, except for a small upper region, demonstrating incomplete chromatin degradation (arrow). Upper left corner of [ **F**] corresponded to the boxed area of the insert and showed H2B specific label in mitotic chromosome of the tumor cell (brown). A layer of collagen fibers (cf) separated that cell from the elongated fibroblast (yellow) with unlabeled nucleus (upper right corner) of [ **F**]. Arrow in the insert points to a condensed mitochondrion.

Most tumor cells displayed ultrastructural features characteristic of hypoxia, i.e., mitochondrial changes and dilated endoplasmic reticulum (ER) cisternae without ribosomes. In some locations hypoxic tumor nodules were breaking apart via prominent anoikis (loss of attachment between cells ^48^) with abundant nano-tentacles. Commonly, cells located next to each other had mitochondria changing in opposing ways. They were either losing their internal cristae without shrinking and thus generating electron lucent vacuoles (seemingly empty or containing whorled membranes that might be intermediate stages of the internal membranes degradation) or becoming smaller and electron dense ([A–C] in Figure 2 & Figure S2). The first type of the morphological changes of mitochondria had been shown to occur as a result of genetically simulated hypoxia followed by necrosis ^49^. The second type at first resembled appearance of mitochondria during mitosis and later, they were indistinguishable from the dark granules in erythroblasts ([D & F] in Figure 2 & Figure S2) and consistent with published images of peroxisomes ^50– 53^. Such opposing changes occurring simultaneously in cells sharing the same microenvironment suggested different fates for them. The one with initiated necrosis could potentially recover when the other had completed its conversion into erythrosome(s). That is because erythrosomes are capable of secreting anaerobically generated ATP ^54^. Oxygen is not critical for erythrosomes themselves because they do not have mitochondria to use it. Initially, electron dense regions of tumor cell nucleus contained chromatin in both cases. However, that changed with the progression of the erythrogenic conversion when detecting histone H2B simultaneously exposed electron dense regions of the nucleus that did not contain chromatin ([E] in Figure 2 & Figure S2). Iron accumulation could be a good alternative reason for such increased electron density not attributable to chromatin condensation. The fibroblasts that encapsulated the tumor were of host origin, similar to the host connective tissue "membrane" encapsulating orthopedic implants ^55^. The GFP-labeled mitotic chromosomes identified the tumor cell whereas the unlabeled fibroblast, on the other side of the collagen layer separating the two, must have been of host origin ([F] in Figure 2 & Figure S2). Together, the host fibroblasts and the encapsulated tumor-derived blood elements created the capsular vaso-mimicry that morphologically resembled veins ([G-I] in Figure 1 & Figure S1). The tumor cell population was clearly heterogeneous; therefore, it could survive by some cells feeding on others, initially via lactic acid secretion ^56^ and then via erythrogenic autophagy ^41^. In this way, the metabolic requirements of the encapsulated tumor nodules appeared to be responsible for initiating the capsular vaso-mimicry.

**Figure 3.**
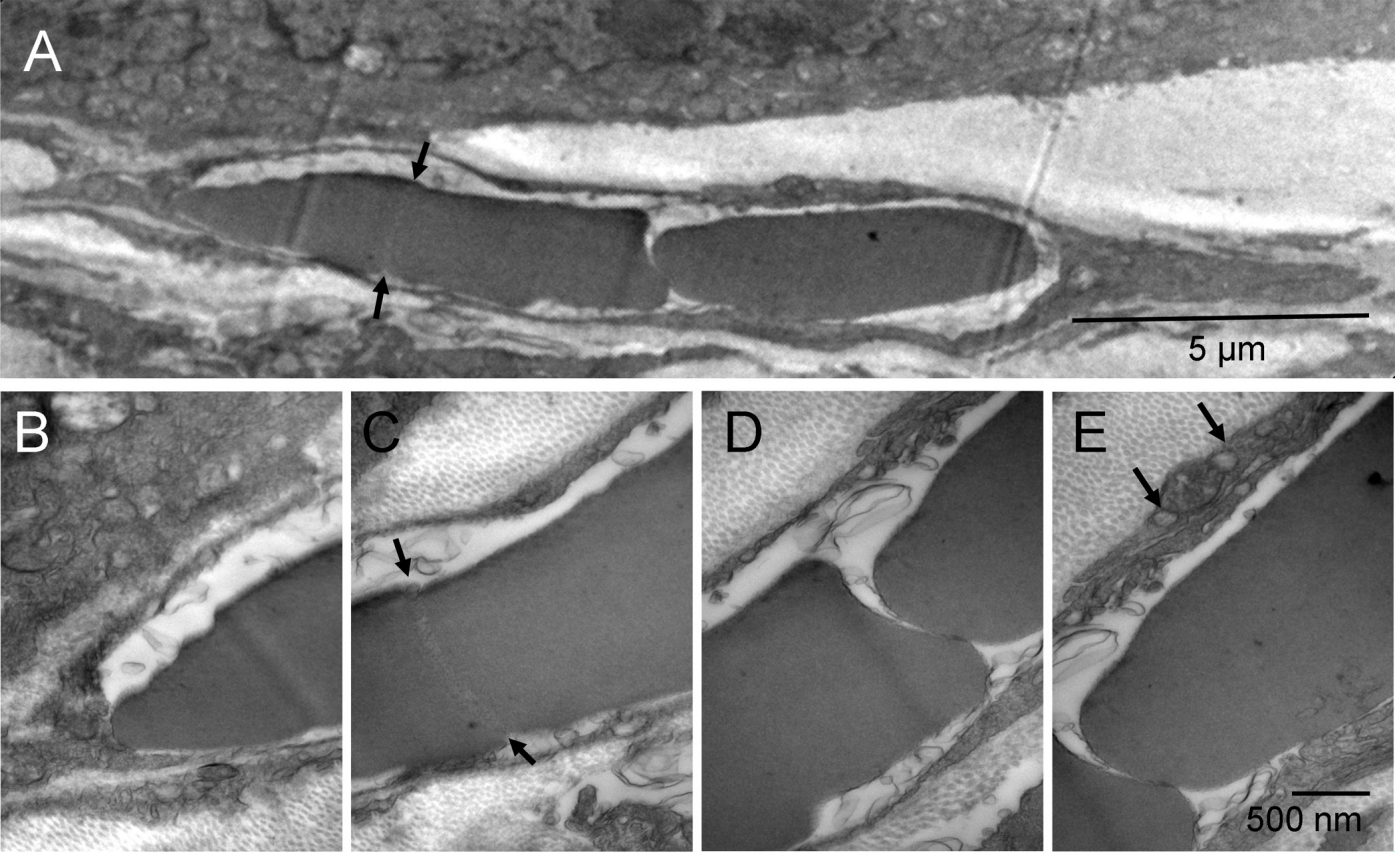
Erythrogenic autophagy in elongated cell between tumor nodules; resemblance to the vasculogenic mimicry. Consecutive splitting of an erythroschizont (red-splitting-body) into erythromers (erythrosomes); one fragment completely separated and the rest in the process of splitting, all surrounded by leftover cytoplasm of the cell producing them [ **A**]. Four enlarged regions of that cell [ **B**– **E**]. A distinct region of the erythroschizont indicated location of the next, already initiated, split (arrows) [ **A** & **C**]. Vesicles on both sides of the mitochondrion contained collagen fibers (arrows) [ **E**].

**Figure S3.**
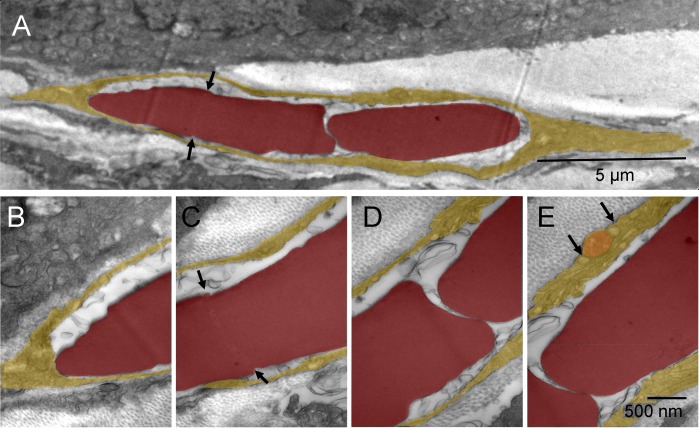
Erythrogenic autophagy in elongated cell between tumor nodules; resemblance to the vasculogenic mimicry. Consecutive splitting of an erythroschizont into erythrosomes (red); one completely separated and the rest in the process of splitting, all surrounded by leftover cytoplasm of the cell producing them (yellow) [ **A**]. Four enlarged regions of that cell [ **B–E**]. A distinct region of the erythroschizont indicated location of the next, already initiated, split (arrows) [ **A** & **C**]. Vesicles on both sides of the mitochondrion (orange) contained collagen fibers (arrows) [ **E**].

Not all tumor nodules were successfully encapsulated at the time of tissue harvest. In some regions, the fibroblasts appeared trapped between possibly faster-growing tumor nodules and, commonly, such fibroblasts were undergoing the erythrogenic autophagy as well. Typically, their cytoplasmic remnants were still present between the erythrosomes and tumor cells. The elongated cells like the one shown between the tumor nodules ( Figure 3 & Figure S3) had large nuclei undergoing conversion into erythrosomes and sparse, metabolically active cytoplasm generating energy and synthesizing protein. What appeared in two-dimensional image as a single file of erythrosomes between tumor nodules was not "a rouleau of circulating erythrocytes" ^18^.

**Figure 4.**
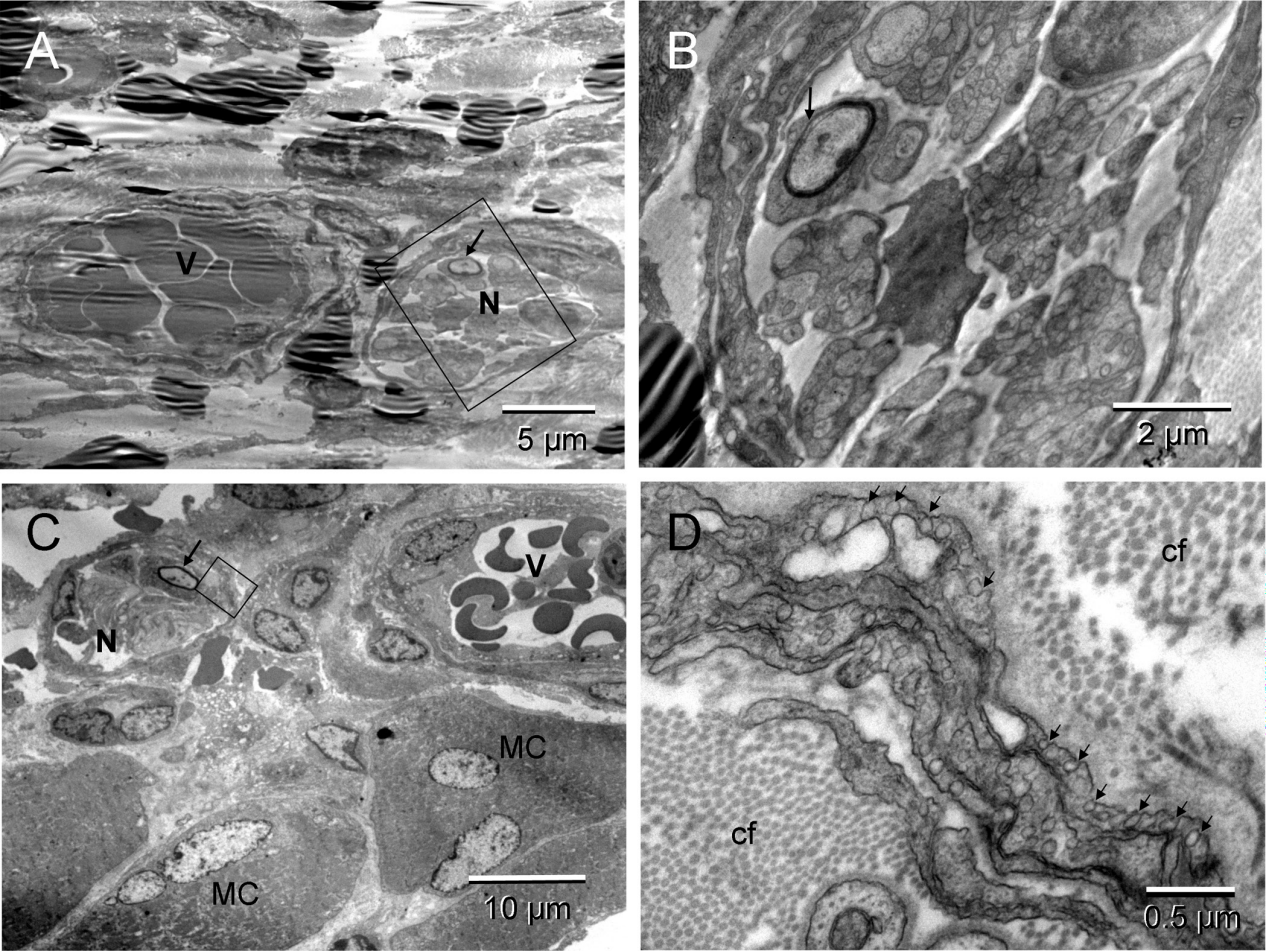
Coordinated morphogenesis of blood vessels (V) and peripheral nerves (N). Two neuro-vascular pairs. The upper pair was less mature; the boxed area of the nerve in [ **A**] (enlarged in [ **B**]) showed incomplete myelination of the neuron (arrows). The pair located next to the muscle cells (MC) [ **C**] appeared more mature; the myelination of one neuron was completed (arrow). Morphology of the erythrosomes surrounded by endothelium was also more mature in [ **C**]. The boxed area, enlarged in [ **D**], featured abundant caveolae (arrows) of the multilayered neurothelium and difference in thickness of the collagen fibers (cf) between those in endoneurium (thinner) and in surrounding ECM (thicker) [ **D**].

**Figure S4.**
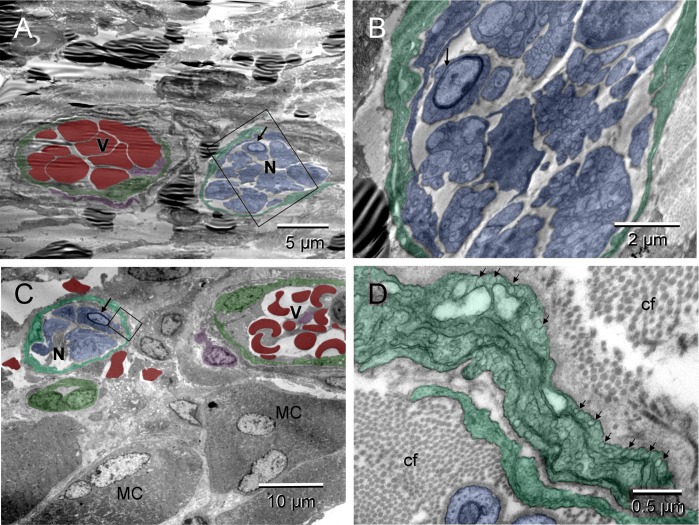
Coordinated morphogenesis of blood vessels (V) and peripheral nerves (N). Two neuro-vascular pairs. The upper pair was less mature; the boxed area of the nerve (blue) in [ **A**] (enlarged in [ **B**]) showed incomplete myelination of the neuron (arrows). The pair located next to the muscle cells (MC) [ **C**] appeared more mature; the myelination of one neuron was completed (arrow). Morphology of the erythrosomes (red) surrounded by endothelium (green) was also more mature in [ **C**]. The boxed area, enlarged in [ **D**], featured abundant caveolae (arrows) of the multilayered neurothelium (emerald) and difference in thickness of the collagen fibers (cf) between those in endoneurium (thinner) and in surrounding ECM (thicker) [ **D**].

The non-malignant tissue repair included formation of new blood vessels and peripheral nerves ( Figure 4 & Figure S4). Developing vessels and nerves arranged in heterotypic pairs suggested a coordinated regulation of their morphogenesis ( Figure 4 & Figure S4). In some regions ECs converting into erythrosomes (hemogenic endothelium) were also seen (Figure 2 in ^57^).

**Figure 5.**
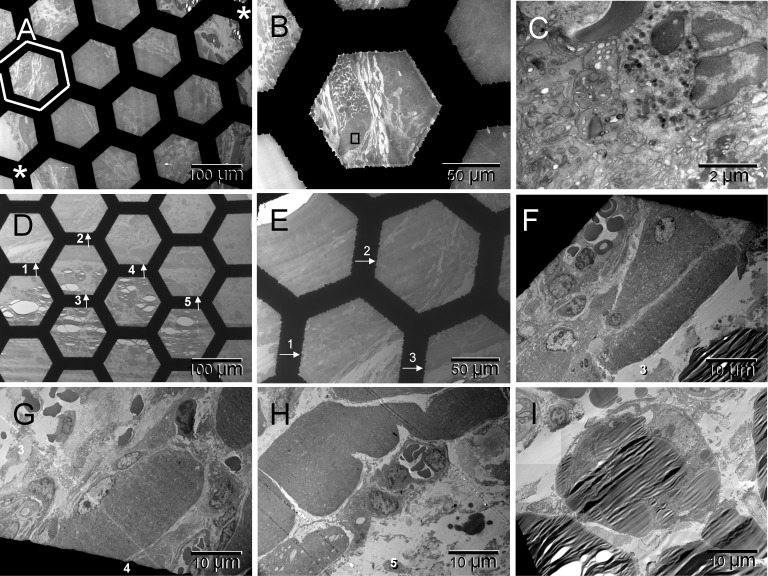
Histomorphology of the spheroid-derived tumors and the surrounding host tissues. The stars in [ **A**] mark edges of the tumor facing glass of the chamber and the host tissues: muscles and fat underlying the host’s dorsal skin (lower left and upper right respectively). The field framed with white hexagon and enlarged in [ **B**] contains the capsular vaso-mimicry separated from the larger tumor mass with a multi-cellular layer of fibroblasts. Fragments of that field are shown at higher magnifications in Figure 1 [ **I**] and here in panel [ **C**] where a cluster of platelets can be recognized next to a neutrophil (left and right sides of the image, respectively). A larger region of the host tissues between the tumor and skin is shown in [ **D**]. Fields 1–3 are enlarged in [ **E**]. [ **F**– **H**] focus on muscle cells from fields 3–5 accompanied by vessels, nerve [ **G**] and lipid droplets [ **F** & **H**]. The adipocyte in [ **I**] is from a region not shown at low magnification. None of the hexagonal fields enclosed by the grid bars was completely filled with the tumor cells indicating that the size of individual nodules roughly matched the oxygen diffusion range (100–200 µm ^58^).

At the time of tissue harvest the histomorphological features of the transplanted tumor spheroids resembled those of spontaneously grown tumors ( Figure 5). The malignant cells were arranged into small nodules surrounded with fibroblasts, vessel-free erythrosomes and some undifferentiated migrating cells (mesenchymal cells). The nodules were not larger than the oxygen diffusion range (100–200 µm ^58^). A thicker fibrotic capsule surrounded the larger clusters of the small nodules. The underlying host muscle cells appeared normal whereas adipocytes were commonly replaced with lipid droplets.

## Discussion

### Surviving without vasculature

Survival of the ectopically implanted breast tumor cells for three weeks without support of host circulatory system was possible due to the erythrogenic autophagy ^41^. The tumor ecological niche resembled a *perpetuum mobile* in its ability to survive without blood vessels. There was a turnover of tumor cells; they kept proliferating and succumbing to erythrogenic autophagy. The system was not really isolated because it used metabolites from the microenvironment, but depending solely on diffusion for that purpose, the tumor could not increase its size. Non-vasculogenic tumors do not grow over several weeks although the tumor cells keep proliferating at rate similar to that of vasculogenic tumors; "They have no or non-functional vessels" ^59^. Those "non-functional" vessels could have been non-circulating erythrosomes, most likely derived from the tumor cells. That was, in fact, another experimental result demonstrating that some tumor cells could survive at the expense of the others.

The relevance of the variable metabolism within a single tumor nodule was that tumor derived erythrosomes might indeed extend viability of adjacent tumor cells by supplying them with vital energy in absence of vasculature. Chromatin degradation products contained all the elements needed to make hemoglobin except iron. Therefore, a cellular conversion into erythrosomes during the neo-hematopoiesis would require soliciting iron from outside the tumor. Indeed, in tumor bearing mice the accumulation of iron was reported to shift from the spleen and liver to the tumor site ^60^. Hemoglobin has evolved to bind oxygen cooperatively, i.e., most efficiently when it is abundant (in lungs where the oxygen concentration is about 100 torr) and gradually less and less efficiently as erythrosomes move through arteries and veins (in peripheral tissues the oxygen concentration is about 20 torr) ^61^. In tumors experiencing hypoxia, one would expect the binding of oxygen to hemoglobin to be least efficient, so the erythrosomes would not compete for oxygen with the tumor cells.

The initial host response to tumor nodules by encapsulating them with fibroblasts was simultaneous with the response to surgical injury caused by the implantation. During the repair process, as the layer of connective tissue grew thicker beneath the glass wall of the chamber, it too was forming new vasculature. That finding showed similarity between the cellular mechanisms of vasculature morphogenesis in growing malignant and non-malignant tissues. Hypoxia, the common metabolic denominator, could force erythrogenesis upon different types of cells, including differentiated ones for example ECs ^57^, if the condition persisted. The potential to convert into erythrosomes was not limited to erythropoietic lineage derived from myeloid precursors. Understandably so, given that the inducing factor, hypoxia, affected the most fundamental function of living cells, i.e., the respiration, generating vital energy aerobically. Under hypoxia, they all had only one alternative to extend their existence, namely, anaerobic generation of energy indispensable to sustain life. That metabolic requirement being shared by all cells experiencing hypoxia imposed formation of similar ultrastructural features on all of them (convergence). Therefore, knowing what the cells do and how they do it regardless of cell lineages is important to control tumor growth. Unfortunately anaerobic metabolism is not unique to tumor cells; others are neutrophils and muscle cells, precursors of erythrosomes in bone marrow, and any dividing cells at mitosis ^57^.

### Vasculogenic switch

The microenvironment determined the fate of tumor cells in a way similar to controlling the fate of other cells. The interactions were mutual and ever changing. The unrelated cells could become similar enough to act as "relatives". In other words, the heterologous environment did not kill the transplanted tumor but gradually the exogenous tissue acquired the ability to engage in the paracrine dialog with local TSCs, (perhaps by acquiring proper cell adhesion molecules ^62^) or the tumor activated its own SCs (CSCs). Trans-differentiation of tumor SCs into ECs was observed here and also reported earlier in glioblastoma ^14, 63– 65^. When that happened, the dormant tumor underwent a vasculogenic switch ^66^. If the process was slow enough, it might not be completed within life span of the host and such tumors would be unnoticed due to their small size, limited by 100–200 µm oxygen diffusion range ^58^. Reported dormant tumors had < 1 mm “diameter”, possibly including fibroblastic coats and necrotic centers ^66^.

### Capsular vaso-mimicry as cellular mechanism of metastasis

Two cellular mechanisms normally beneficial to the organism when acting independently, one involved in tissue nourishing and the other in healing, i.e., erythrogenesis and scar formation (or foreign body encapsulation ^55^) respectively, became deleterious by creating the capsular vaso-mimicry when they coincided in the ectopically implanted tumor. The newly emerged vessel-imitating structures contained cells of tumor and host origin. They did not contain circulating blood initially, but could potentially fuse with the morphologically similar host lymphatic vessels or veins. If the conversion of the tumor cell population into erythrosomes were incomplete at the time of the merger, the fusion would facilitate metastasis. The anastomosis with lymphatic vessels might be more likely than with blood vessels (particularly arteries) because the former are comprised of a single endothelial cell layer, have no pericytes and only incomplete basement membrane. That would be consistent with the observation that metastases of most cancers occurred initially through the lymphatics ^67^. The dissociation of tumor cells from one another, i.e., anoikis ^68^, commonly seen in necrotic regions, might be due to hypoxic stress and starvation. That way each cell would gain direct access to interstitial fluid and the cell surface would greatly increase through multiple, meandering nano-tentacles that appeared to be an early sign of stress. At the same time, the loss of attachment to other cells could facilitate their dissemination by breaking tumor tissue into small cell clusters or single cells that could be carried away by blood flow. Whereas converting a fraction of the growing tumor population into erythrosomes solved the immediate energy metabolism problem for the rest of the population temporarily, the capsular vaso-mimicry could assure a future steady supply of new energy resources in the end.

Concerning the prospect of controlling initiation of metastasis, the cellular mechanism presented here appeared more manageable because it did not depend on great biological diversity of primary tumors. The initiation of capsular vaso-mimicry was governed by metabolic requirements rather than the genetic repertoire of the tumor. Clusters of the primary tumor cells could be passively carried to different tissues by blood flow and become immobilized when they reached vessels narrower than their own dimensions, in a tissue non-specific manner. However, the fate of such randomly dispersed metastatic tumor "seeds" would depend on their phenotypic compatibility with the local "soil" ^69^. Thus, the vasculogenic switch could occur in the secondary locations either immediately or after a variable period of latency depending on the initial degree of relevant similarities. Liver being formed relatively early during embryogenesis and later maintaining primitive vasculature might be most compatible with tumors for that reason and therefore most prone to metastases, as observed clinically and shown experimentally ^70^. If the metastasized tumors did not adjust their properties as needed to establish paracrine dialog with local TSCs, they would stay dormant. The dormancy would not necessarily be permanent because surviving via erythrogenic autophagy was accompanied by proliferation ([F] in Figure 2 & Figure S2) and therefore equipped the tumor with a source of the biological diversity.

The concept of the distant niche anticipating an invader and getting ready for it ^71, 72^ could be adopted as follows. Dissemination of tumor cells through circulating blood could occur due to capsular vaso-mimicry targeting all organs but a successful metastasis would only develop in tissues somewhat similar to the one where the primary tumor developed. This would be consistent with the seed and soil theory ^69, 73, 74^. On the other hand, if the tumor was large enough to produce meaningful levels of cytokines and growth factors in circulating blood, the effect on un-invaded homologous tissues should be comparable to that caused by a smaller number of tumor cells that have metastasized. That is how anatomically distant but phenotypically compatible tissue could become activated by the tumor before metastasizing cells got there.

The postnatal extramedullar erythropoiesis at the location other than bone marrow ( *medulla ossea*) was observed previously in spleen ^75– 78^ and adipocytic tissues ^79^. However, in our model the local host adipocytes responded to the tumor implantation with the lipogenic rather than the erythrogenic autophagy. The entire cells were converted into lipid droplets. If the tumor cells could be treated to re-direct their metabolism to lipogenesis instead of erythrogenesis, perhaps the metastatic potential of the capsular vaso-mimicry could be abolished and ultimately the entire tumor replaced with fat. A non-malignant type of undifferentiated cells, human mesenchymal stem cells, can accumulate lipid under hypoxia, although normally they would differentiate along several pathways to form bone, cartilage, tendon, muscle or adipose tissues. In that case the potent lipogenic effect of hypoxia was independent of PPAR-γ2 maturation pathway ^80^.

### Vasculogenic mimicry

The structures shown in Figure 3 & Figure S3 and the earlier reported vasculogenic mimicry ^15^ could be of the same nature. The remnants of cells that produced erythrosomes could be responsible for PAS staining due to their glyco-lipid components and, more importantly, for fusion with capillaries of main circulatory system, at stages later than analyzed here. Our tumors were significantly smaller ("diameter" of < 1 mm) than those described in the literature (1 cm or more). Lack of hierarchy in the network pattern of the aggressive tumors suggested a lack of blood circulation. However, small molecules used to study intra-tumoral microcirculation by injecting a dye into a vessel located close to it could rapidly diffuse through such spaces ^17, 18^. If blood were circulating through the vascular mimicry, there should be no problem with drug delivery to such tumors. Therefore, that kind of mimicry is probably a form of erythrogenic autophagy of fibroblasts associated with the presence of metastatic tumors. The metastases could have been initiated via capsular vaso-mimicry earlier, when the primary tumor nodules were small.

### Leakiness of tumor vessels

The distance between capillaries in tissue sections suggested that, within the 100–200 µm zones, cell membranes did not present a barrier for diffusion of nutrients as well as oxygen. On the other hand, toxic metabolic products can be sequestered into intracellular vesicles to protect the cytoplasm. Although indistinguishable by TEM, the bi-layer lipid membranes vary with regard to their molecular composition. The leaky outer cell membranes permit passage of small molecules whereas the more selective inner vacuolar membranes provide a mechanism for intracellular compartmentalization. Considering what we now know about cytoevolution leading to ECs differentiation ^41^, one could make a premise that luminal surface of the polarized endothelial cell was a functional equivalent of the inner membrane. Therefore, it could present a barrier preventing uncontrolled diffusion of some molecules. Caveolae would serve as a compensation for such an indiscriminate barrier and would provide a structural basis for selective (controlled) transport across the membrane ^81^. That might be why ECs have them in great numbers and the absence of caveolae in brain endothelium correlates with the functional blood-brain barrier ^82^. Vascular lumen in that context would be a functional equivalent of intracellular vesicle. One could conclude that host fibroblasts encapsulating the tumor and creating the capsular vaso-mimicry by positioning themselves around erythrosomes should not present a barrier for the diffusion process because they did not go through the process of cytoevolution resulting in polarization of outer cell membrane into luminal and abluminal. Morphologically, the tumor capsular vaso-mimicry resembled lymphatic vessel or vein, however it was neither. It had fibroblasts in place of ECs, therefore diffusion across the walls of the capsular vaso-mimicry (and further) would not be restricted. That could be a new explanation for the leakiness of the tumor pseudo-vessels, whereas in genuine tumor supporting vessels, control of permeability would remain tight.

### Spectral *in vivo* oxygenation

The new understanding of the cellular mechanisms involved in the tumor neovasculature formation provokes some additional retrospective thoughts on earlier published results regarding vasculature related issues that were also based on the model of breast tumors grown in the mouse skin fold chamber. For example, abnormal microvascular oxygen transport demonstrated by spectral imaging of hemoglobin saturation ^83, 84^. Anastomoses between vessels with significantly different oxygenations could be explained by the fusion of hypoxic capsular vaso-mimicry with a vein containing circulating blood; the direction of the flow initiated that way would be expected to be the same as in the vein involved in the fusion, as observed (Figure 8 in ^84^). The resulting larger hybrid vessel would initially have a flattened profile, as seen in malignant neurilemmoma grown in a hamster cheek pouch chamber ^85^. What looked like "acute local stoppage of blood flow" could correspond to the lack of the flow in the vascular vaso-mimicry before the anastomosis ^84^. Shunting of inspired oxygen into tumor venules, presumed to occur due to arterio-venous anastomoses (Figure 10 in ^83^) could alternatively be explained by stable saturation of hemoglobin located in non-circulating erythrosomes within tumor capsule as well as within the regions mimicking vessels ( Figure 1 & Figure S1). The oxygen could have remained bound to hemoglobin if the surrounding cells did not have structurally sound (functional) mitochondria. We have shown that mitochondria of tumor cells in the microenvironment of such stagnant erythrosomes were structurally impaired (converted into peroxisomes or necrotized) and accompanied by calmyrites. Such ultrastructural features are consistent with anaerobic metabolism. In the absence of functional mitochondria, the tumor cells would have no use for the abundant oxygen. Consequently, it should not be surprising that increased oxygenation of breast adenocarcinoma by treatment with, for example, darbepoetin alpha, had no desirable effect on the tumor’s responsiveness to radiotherapy ^86^. Oxygenation was probably increased in the erythrosomal component of the tumor, not in the tumor cells (a distinction impossible to make by clinical radiology).

## Conclusions

The results demonstrated that a balance between tumor growth and formation of its own vasculature could shift reversibly as dictated by a changing microenvironment. *In vitro*, where proper atmosphere and nutrients were available, tumor cells did not need vasculature and none formed. That changed when they found themselves back in the live mice but not connected to the host vasculature. Hypoxia forced some tumor cells to change their energy metabolism to an anaerobic pathway. That way, they could salvage the remaining tumor cells in two ways: by secreting lactic acid ^56^ or ATP ^54^ (similarly to muscle cells and erythrocytes, respectively) and by initiating the vaso-mimicry. Time gained by the metabolic switch allowed for triggering the genuine vasculogenic switch and exposed self-organizing potential of the malignant tissue (limited to formation of its own vasculature). Creating the capsular vaso-mimicry would require sufficient numbers of cancer cells in the initiating nodule. While some tumor cells were evolving into erythroblasts and megakaryocytes and inducing differentiation of ECs, others kept proliferating. Such activation of differentiation in some cancer cells was consistent with the organoblasts concept ^41^. By definition they could be referred to as cancer stem cells (CSCs). However, alkaline phosphatase cannot be used as a marker specific for CSCs because it was detected in erythroblasts ^87^. If the enzyme plays a role in degradation of chromatin during the erythrogenic conversion of erythroblasts it could be associated with growth of any tissue, not only malignant.

Eventually, the heterologous host TSCs also engaged into paracrine dialog with the tumor (via cytokines and growth factors ^71^). The distinction between the two sources of SCs was based here on the location where early stages of vasculature formation were seen within the tumor capsule or next to it ( Figure 1 & Figure S1). Whereas the existence of somewhat controversial CSCs ^28, 88^ was exposed in the ectopic environment, it was masked by availability of homologous TSCs in the pseudo-orthotopic one ^41^. Such interpretation regarding the role of homologous TSCs in neo-vasculature morphogenesis was consistent with earlier reports stating that not bone marrow derived EC precursors ^89, 90^ but TSCs from tumor microenvironment differentiated into vasculature that supported tumor growth ^91^. They were also shown to support vascular nonmalignant engraftment ^92^.

On the other hand, after pseudo-orthotopic implantation, TSCs from grafted breast tissue formed vasculature for the breast tumor sooner because malignant tissues maintained some characteristics of their tissue of origin. The two related cell types were immediately ready to cooperate in executing the tissue self-organizing potential ^41^. The source of SCs that generated tumor vasculature under different circumstances (tumor, host or grafted homologous tissue) mattered with regard to how soon the vasculature formation could begin. However, in each case hematopoiesis supporting the growing tissues was extramedullar. That observation was new and suggested a physiological role for the aerobic glycolysis (the Warburg phenomenon) in tissue morphogenesis, as addressed elsewhere ^57^.

## Figures and Tables

**Figure S5.**
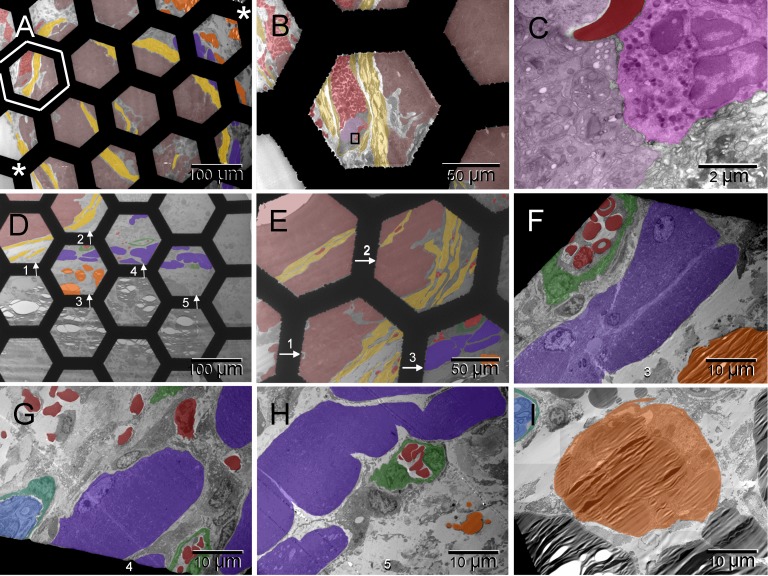
Histomorphology of the spheroid-derived tumors and the surrounding host tissues. The stars in [ **A**] mark edges of the tumor (brown) facing glass of the chamber and the host tissues: muscles (dark purple) and fat (orange) underlying the host’s dorsal skin (lower left and upper right respectively). The field framed with white hexagon and enlarged in [ **B**] contains the capsular vaso-mimicry separated from the larger tumor mass with a multi-cellular layer of fibroblasts (yellow). Fragments of that field are shown at higher magnifications in Figure 1 [ **I**] and here in panel [ **C**] where a cluster of platelets can be recognized (light purple region on the left) next to a neutrophil (pink on the right). A larger region of the host tissues between the tumor and skin is shown in [ **D**]. Fields 1–3 are enlarged in [ **E**]. [ **F**– **H**] focus on muscle cells from fields 3–5 accompanied by vessels (green), nerve (blue) [ **G**] and lipid droplets (orange) [ **F** & **H**]. The adipocyte in [ **I**] is from a region not shown at low magnification. None of the hexagonal fields enclosed by the grid bars was completely filled with the tumor cells indicating that the size of individual nodules roughly matched the oxygen diffusion range (100–200 µm ^58^).
