# Supplementary material for: II. Capsular vaso-mimicry formed by transgenic mammary tumor spheroids implanted ectopically into mouse dorsal skin fold: implications for cellular mechanisms of metastasis
Source: F1000Res. 2013 Apr 23;2:9. Originally published 2013 Jan 10. [Version 2] doi: 10.12688/f1000research.2-9.v2 (PMC3869488; doi:10.12688/f1000research.2-9.v2)
Supplement: Peer review history

# Peer review: Referee response for version 2

**Published:** 2013-06-11

**Authors:** Anita Bandrowski

**Affiliations:** 1 Division of Biological Sciences, University of California, San Diego, La Jolla, CA, USA

This report entitled “II. Capsular vaso-mimicry formed by transgenic mammary tumor spheroids implanted ectopically into mouse dorsal skin fold: implications for cellular mechanisms of metastasis” by Witkiewicz *et al*., complements and enhances the other papers by these authors, helping to elucidate the findings in the other papers and as such should be published concurrently.

There are several important points touched on by the authors. One, the malignant cells form their own erythrosomes and later blood vessels. Two, these erythrosomes have a different metabolic need than other cells in the region, which become the food. Three, the 3 week time point, which is described in this study, and the set of processes that governs this transformation of tumor cells into erythrosomes/proto blood vessels based on fibroblasts is an important key to the subsequent presumed metastatic events. This is a very interesting group of insights and if for no other reason than to test further this hypothesis, this paper should be published. 

This reviewer agrees with reviewer #1 and #2, who point out that the experiments would be more robust if further experiments would be done to gather data at various time points or from different models. This is a fair point, and I too believe that more time points and different models would benefit this work however the current work, in all three papers being concurrently published represents a significant work. Perhaps the authors can address whether such follow up work, i.e., to increase the time points and to try this with other cell lines, is on-going.

The authors point out other interesting findings including the following sentence “The underlying host muscle cells appeared normal whereas adipocytes were commonly replaced with lipid droplets.” Suggesting a mechanism by which tumors can feed on the host cells in ways predicted by epidemiological findings relating various cancers to obesity. If true, this would begin to elucidate a causal mechanism with the current correlation between some of the epidemiological findings. However the authors don’t really describe this point and it would be of benefit to do so.

I have read this submission. I believe that I have an appropriate level of expertise to confirm that it is of an acceptable scientific standard.

# Peer review: Referee response for version 2

**Published:** 2013-05-30

**Authors:** Karolina Kucharova

**Affiliations:** 1 Sanford-Burnham Medical Research Institute, La Jolla, CA, USA

The title: “II. Capsular vaso-mimicry formed by transgenic mammary tumor spheroids implanted ectopically into mouse dorsal skin fold: implications for cellular mechanisms of metastasis” does not seem to be appropriate, because authors did not observe metastasis in their study. 

The authors observed the collagen-producing fibroblasts encapsulating the tumor spheroids that led to a form of vaso-mimicry in the subcutaneous environment. The authors speculate that such capsular vaso-mimicry could potentially facilitate metastasis by fusing with morphologically similar lymphatic vessels or veins. I think that the authors’ opinion that vaso-mimicry may be involved in tumor metatstasis is well presented in their abstract, but this speculation shouldn’t be presented in the title. In the abstract, I suggest to remove the text “co-implantation of lactating breast tissue created stimulating microenvironment “, because this study is not focusing on finding in the microenvironment of lactating breast tissue.  

Did the authors observe lymphatic vessels in close proximity to structures similar to the vasculogenic mimicry which contained non-circulating erythrosomes formed between tumor nodules? Did authors observe the different cells, e.g. pericytes, macrophages etc. in their study? If yes, it will be valuable to add into the abstract and results section. 

The methods are well documented, except there is little information about the chamber and how cells were implanted into the chamber. Better descriptions of techniques can help to readers to understand why authors studied tumor spheroids implanted ectopically into mouse dorsal skin fold.

In the results section (8th sentence: “Occasionally some blood cells were in close contact with supporting nucleated cells “), - I suggest being more specific which type of blood cells and supporting nucleated cells were observed in their study and rewrite this sentence. 

I suggest adding arrows, arrowheads and other symbols to the figures, figure legends, and text to indicate exactly which cells are being discussed. Or the authors may switch the B/W Figure 1 with a color Figure S1. This type of correction should be carried on to all other figures.

I have read this submission. I believe that I have an appropriate level of expertise to confirm that it is of an acceptable scientific standard, however I have significant reservations, as outlined above.

# Peer review: Referee response for version 2

**Published:** 2013-05-02

**Authors:** Ygal Haupt

**Affiliations:** 1 Tumour Suppression Laboratory, Peter MacCallum Cancer Centre, East Melbourne, VIC, Australia

The major response of the authors was the inclusion of new images and the addition of the word 'implication' to the title of the manuscript. While these two additions improve the manuscript, the major issues that were raised remained. These include the basis of the study is on *in situ* ultra structural analysis; the title does not correctly reflect the conclusions from this paper; the lack of metastatic response and hence the lack of a cellular mechanism for metastasis, and the use of one mouse model and one cell line. 

The authors provide a lengthy response arguing that the TEM complements other methods used elsewhere in earlier publications as their response. They further claim that others have also used a single mouse model and a single cell line, without specific citations. While this may well be the case in other studies providing detailed mechanism, this claim by the authors does not address the limitation of the current study as raised by us and the other reviewer previously.

I have read this submission. I believe that I have an appropriate level of expertise to confirm that it is of an acceptable scientific standard, however I have significant reservations, as outlined above.

# Peer review: Referee response for version 1

**Published:** 2013-04-24

**Authors:** Maria Vinci

**Affiliations:** 1 The Institute of Cancer Research, Belmont, Sutton, Surrey, UK

The authors describe an interesting model that involves the ectopic implantation of tumour spheroids (N202 breast tumour cell line) into the mouse skin fold chamber. By using high quality ultra structure images, the authors describe tumour survival to hypoxia and formation of capsular vaso-mimicry as the event that determine the vasculogenic switch, 3 weeks after implantation and that facilitate metastasis. 

Although the work is valuable for the novelty of the process described, on the other side, it also presents some issues, for which I have reservations. The work is based only on one tumour model, the number of animals used for some of the observations is too low, the analysis is performed only by *in situ* ultrastructure and no quantitative data are provided as well as no information on the number of fields of view/slide analysed. 

Also the images shown are all in very high power magnification. It would be useful to include images at lower power magnification to have a better overview of the surrounding tissue. Also, the authors do not provide any evidence of tumour metastasis, or tumour dissemination occurring from the original implantation, but imply that the observed vaso-mimicry process could facilitate metastasis. The authors should have looked at the process at different time points: earlier and later than the 3 weeks time point selected for the study. 

Minor comments: the article is in general too wordy with the results often containing discussion points. Typos: in the title "ectopicly" should be "ectopically" and on page 9, "phenotipicly" should be "phenotipically".

I have read this submission. I believe that I have an appropriate level of expertise to confirm that it is of an acceptable scientific standard, however I have significant reservations, as outlined above.

# Response

**Published:** 2013-04-23

**Authors:** Halina Witkiewicz

**Affiliations:** PRISM, USA

**Magnification**

The article has been amended to include examples of images taken at lower power magnification. Figure 5 shows how the overall topography of each section was examined before focusing on the most interesting regions.  

**Implications for tumor metastasis**

In the title of the amended article we have added the word “implications” to reflect the distinction between the new results and their implications more precisely. In other parts of the article that distinction is clear. The original findings (including the blood elements and tumor cells inside the vessel-resembling structure, i.e., the capsular vaso-mimicry) are described in the Results section whereas the implications derived from those data and their significance are placed in the Discussion section. For the reader’s work such implications may be most useful when they represent new ideas and envision new directions for the future research. Without hinting in the second part of the title on significance of the new findings some readers could miss the article despite its relevance to their studies. 

***In situ*** **ultrastructure only**

The issues addressed in our study are central in tumor biology and have been approached in many ways. However, without the type of validation possible by the *in situ* analysis of a particular living system at high resolution, the published work left many questions unanswered and created multiple controversies. Yet, our in depth analysis of the TEM images would not have been possible without the other approaches. We drew freely from the abundant experimental data accumulated in the literature regardless of the method used to generate them. The visual information from our work inspired logical connections between various kinds of earlier published data, often derived from various sources. Therefore, our conclusions and hypotheses do not rely solely on TEM. Our approach fills up the essential void; it “connects the dots”. The approach and the model we used are the strength of our study (as discussed in article III). 

The tissue ultrastructural analysis *in situ* brings together aspects of tumor biology traditionally treated independently: hematopoiesis, vascular biology, tumor metastasis and metabolism. **Such exposure of the interdependence among those aspects introduces the tissue level into the general conversation on tumor biology at cellular & molecular levels. What a single experimental approach is depends on the definition. One can base the criteria on instruments used or on targets analyzed. Bridging the tissue and molecular levels, we used TEM to characterize tissues, cells, organelles and specific proteins *in situ *(immunocytochemistry). We used a genetically engineered, GFP-labeled cell line and the graft-donor mice. Intra-vital light and fluorescent microscopy helped to monitor the rate of tumor growth *in vivo*. The future belongs to this sort of combination of methods, i.e., the use of ultrastructural analyses *in situ* to validate other methods. 

**One tumor model**

Even though our initial observations have been limited to the breast cancer model, there are some indications that the capsular vaso-mimicry is not unique to that tumor type. As discussed in the article, the structure related to the capsular vasomimicry (Figure 3) was of the same nature as the vascular mimicry first seen in melanoma [ PM: 10487832]. Such mimicry was subsequently found in tumors of ovary [ PM: 11290546; PM: 11839501], prostate [ PM: 11813211; PM: 12359755] and in glioblastoma [ PM: 20375132]. Reporting studies based on one tumor type in one animal model is not unusual. The phenomenon of encapsulating tumors with fibroblasts is common among various types of tumors, although not every encapsulation forms the capsular vasomimicry. The prominent fibrous capsule associated with non-invasive insulinoma in the mouse model of pancreatic islet tumorigenesis did not coincide with the invasive tumor type either [ PM: 12086849]. Unlike the monolayer, a thick layer of fibroblasts encapsulating the tumor did not resemble a vascular wall. Figure 5 in the amended version of the article shows examples of both. 

**Quantitative data & time points**

Our goal was accomplished by generating unprecedented photographic documentation of spontaneous intercellular relations leading to formation of the capsular vaso-mimicry. The nature of the study was exploratory and observational therefore the results are qualitative. The images represented raw data, i.e. a direct demonstration of the phenomena that had actually occurred *in vivo*. The animals were not subjected to any kind of treatment except implantation of the tumor. The conventional thinking in terms of comparing treated and control groups over a period of time to measure an anticipated effect and to evaluate its statistical significance was not applicable here. The time progression was not critical either, as opposed to the embryonal growth and development, because the tumor never matures. *In vivo* the cells were responding to locally variable microenvironment therefore they were not functionally synchronized. Consequently a range of different stages of the observed processes could be found simultaneously within single sections. 

To illustrate our conclusions we selected representative images after careful analysis of many (several hundreds per mouse at various magnifications). One should keep in mind that the tumors were about 1 mm in “diameter” when viewed through a dissecting microscope. The photographed fields were selected, not randomly picked; therefore stating how many they were would be meaningless. We stopped the analysis when the findings became redundant and we understood the observed phenomena. In the literature the number of studied animals tends to be inversely proportional to complexity of assays. For example, one study had animal groups of variable size: n=18 for flow cytometry and n=6 for histology ( PM: 15883207). For electron microscopy studies it is common to use samples from small number of individuals ( PM: 20439620). In all five mice that we used (in this and the accompanying articles) the fundamental cellular mechanism of initiating the vasculature formation turned out to be the same. Thus, the number of animals was adequate for the goal of this particular study. Increasing the number of sacrificed animals above the necessary minimum, just for the number sake, would be superfluous and against the animal welfare rules. 

**Minor comments**

Dealing with images as raw data makes it difficult to find the right balance between describing what they show (and what the readers can see) and what they mean. We have tried our best to justify the interpretation by indicating what criteria were used to identify the presented structures. That often involved including literature references. Some improvements have been made in the amended version of the article. Both forms of the adverbs “ectopicly” & “ectopically” as well as "phenotypicly" & "phenotypically" are used by different sources. Another example of such inconsistency is the pair of words “tumor” & “tumour”. We leave the choices to the editor.

# Peer review: Referee response for version 1

**Published:** 2013-01-21

**Authors:** Ygal Haupt

**Affiliations:** 1 Tumour Suppression Laboratory, Peter MacCallum Cancer Centre, East Melbourne, VIC, Australia

The authors report here on how tumour cells survive in an ectopic environment by implantation of spheroids of N202 breast cancer cells into a mouse dorsal skin chamber. Their major finding is that the implanted tumour spheroids survived for 3 weeks without any support of the host circulatory system.They propose that vasculogenic switch and erythrogenic authophagy of some tumour cells are the key process driving the survival and growth of the tumour cells. They find that tumour cells secrete ECM component and can generate their vasculature by converting into erythrosomes thereby supporting other tumour cells. The authors propose that this process is governed by anaerobic metabolism. The limitations of this study are: 1) the study is based on *in situ* ultra structural analysis of tumour growth and survival. There was no metastatic response or circulating tumours in their model, hence no cellular mechanism of metastasis as the title implies.Further, the study is based on one mouse model and one breast tumour cell line. While the study highlights the importance of anaerobic metabolism in metastasis, this concept has been discussed in the literature.

I have read this submission. I believe that I have an appropriate level of expertise to confirm that it is of an acceptable scientific standard, however I have significant reservations, as outlined above.

# Response

**Published:** 2013-04-23

**Authors:** Halina Witkiewicz

**Affiliations:** PRISM, USA

***In situ*** **ultrastructural analysis**

The issues addressed in our study are central in tumor biology and have been approached in many ways. However, without the type of validation possible by the *in situ* analysis of a particular living system at high resolution, the published work left many questions unanswered and created multiple controversies. Yet, our in depth analysis of the TEM images would not have been possible without the other approaches. We drew freely from the abundant experimental data accumulated in the literature regardless of the method used to generate them. The visual information from our work inspired logical connections between various kinds of earlier published data, often derived from various sources. Therefore, our conclusions and hypotheses do not rely solely on TEM. Our approach fills up the essential void; it “connects the dots”. The approach and the model we used are the strength of our study (as discussed in article III). 

The tissue ultrastructural analysis *in situ* brings together aspects of tumor biology traditionally treated independently: hematopoiesis, vascular biology, tumor metastasis and metabolism. **Such exposure of the interdependence among those aspects introduces the tissue level into the general conversation on tumor biology at cellular & molecular levels. What a single experimental approach is depends on the definition. One can base the criteria on instruments used or on targets analyzed. Bridging the tissue and molecular levels, we used TEM to characterize tissues, cells, organelles and specific proteins *in situ *(immunocytochemistry). We used genetically engineered, GFP-labeled, cell line and the graft-donor mice. Intra-vital light and fluorescent microscopy helped to monitor the rate of tumor growth *in vivo*. The future belongs to this sort of combination of methods, i.e., the use of ultrastructural analyses *in situ* to validate other methods. 

**No metastatic response****

In the title of the amended article we have added the word “implications” to reflect the distinction between the new results and their implications more precisely. In other parts of the article that distinction is clear. The original findings (including the blood elements and tumor cells inside the vessel-resembling structure, i.e., the capsular vaso-mimicry) are described in the Results section whereas the implications derived from those data and their significance are placed in the Discussion section. For the reader’s work such implications may be most useful when they represent new ideas and envision new directions for the future research. Without hinting in the second part of the title on significance of the new findings some readers could miss the article despite its relevance to their studies. 

**One model**

Even though our initial observations have been limited to the breast cancer model, there are some indications that the capsular vaso-mimicry is not unique to that tumor type. As discussed in the article, the structure related to the capsular vaso-mimicry (Figure 3) was of the same nature as the vascular mimicry first seen in melanoma [ PM: 10487832]. Such mimicry was subsequently found in tumors of ovary [ PM: 11290546; PM: 11839501], prostate [ PM: 11813211; PM: 12359755] and in glioblastoma [ PM: 20375132]. Reporting studies based on one tumor type in one animal model is not unusual. The phenomenon of encapsulating tumors with fibroblasts is common among various types of tumors, although not every encapsulation forms the capsular vaso-mimicry. The prominent fibrous capsule associated with non-invasive insulinoma in the mouse model of pancreatic islet tumorigenesis did not coincide with the invasive tumor type either [ PM: 12086849]. Unlike the monolayer, a thick layer of fibroblasts encapsulating tumor did not resemble a vascular wall. Figure 5 in the amended version of the article shows examples of both. 

**The concept**

The capsular vaso-mimicry has not been discussed in the literature and the issue of leakiness of the tumor vessels remains unclear. No mechanism was proposed to make logical connections between the molecular events and the intercellular relations leading to the metastasis via the erythrogenic autophagy. Previously reported correlations between anaerobic metabolism and metastasis were used to predict clinical outcome.
